# Storable, neglected, and underutilized species of Southern Africa for greater agricultural resilience

**DOI:** 10.1002/pei3.70004

**Published:** 2024-08-23

**Authors:** Daniel J. Winstead, Michael G. Jacobson

**Affiliations:** ^1^ Department of Ecosystem Science and Management The Pennsylvania State University University Park Pennsylvania USA

**Keywords:** agrobiodiversity, disaster risk reduction, food resilience, Southern Africa, underutilized crops

## Abstract

The Southern African region suffers from drought and food system uncertainty with increased risks due to climate change, natural disasters, and global catastrophes. Increasing crop diversity with more appropriate and resilient crops is an effective way of increasing food system resilience. We focus on crop species that are native or naturalized to an area because of their increased resilience than those that are not naturally occurring. Additionally, crops that are easily stored are more useful in times of drought and disaster. In this systematic review, we use scientific interest in neglected and underutilized species (NUS) from Southern Africa to help define next steps toward their cultivation and development as a marketable crop. We found that although scientific interest is minimal for storable Southern African NUS, these crops are worth scaling up due to their economic and nutritional value. We outline next actionable steps and specific NUS for production in a more agrobiodiverse and resilient agriculture system.

## 1 INTRODUCTION

Natural and man‐made disasters are becoming more frequent and often result in food system disruptions (Garner et al., 2017; Smith et al., 2022; Winstead et al., 2023). Countries in the Southern African floristic region (defined in this paper as South Africa, Botswana, Namibia, Lesotho, and Eswatini) already suffer from water scarcity and malnutrition to varying degrees, which climate change has and will worsen (de Waal & Whiteside, 2003; Inman et al., 2020; Mukwada et al., 2021; Nhamo et al., 2019; Orimoloye et al., 2021; Temoso et al., 2018). During times of disaster, food and water scarcity put many in Southern Africa at even greater risk of malnutrition and hunger. To reduce current malnutrition rates and increase systemic food resilience, multiple sectors of Southern Africa's agriculture and modes of food access and availability need to change (Mabhaudhi, Chimonyo, & Modi, 2017; Mabhaudhi, O'Reilly, et al., 2016).

One such sector is the realized physical resilience of the agricultural system, in other words, the capacity of the system to deal with such changes in climate which have become so inevitable. Bolstering food system resilience would be beneficial for short‐term disasters and sustainability, and if done correctly, would also bolster the food system to endure even larger shocks such as sun‐blocking global catastrophic risks (GCRs). Disasters such as regional famine would pale in comparison to GCRs like a nuclear war, asteroid strike, or super volcano eruption, which would disrupt most food and energy systems. Many post‐GCR crop models have predicted that even a small‐scale nuclear war between nations would significantly decrease food production across the globe (Denkenberger & Pearce, 2015; Jagermeyr et al., 2020; Winstead & Jacobson, 2022a, 2022b; Xia et al., 2015). Severe climate shifts to a drier, colder, and darker environment after a GCR (e.g., “nuclear winter”) would cause crop production to decline for many years across the world (Coupe et al., 2019; Toon et al., 2007, 2019). Additionally, the annual risk of a nuclear war occurring has been estimated as high as 1%, which could certainly continue to be decreased with more strategic diplomacy (Barrett et al., 2013; Ord, 2020). Both the increased risk of natural disasters and higher risk of GCRs mean that creating resilient and local food systems worldwide is more important than ever.

Although GCRs remain relatively unlikely, current climate change poses similar threats that also require a more resilient agricultural and food system. Although current global warming does not appear to have similar conditions to a post‐GCR climate, a common thread between the challenges of growing food during a GCR, current climate change, and natural disasters is the prevalence of drought and need for drought‐resistant crops. Current trends and models in climate change show increases in drought and desertification in most of Africa, as well as many other regions in the world (IPCC, 2019). Therefore, it is in best interest to create a drought‐resistant agricultural system now to benefit agriculture resilience under current conditions and prepare for probable future conditions.

One important component of building resilient drought‐resistant food systems is increased agrobiodiversity which includes drought resistant crops (Mabhaudhi, Chimonyo, & Modi, 2017; Rosero et al., 2020; Winstead et al., 2023; Winstead & Jacobson, 2022a). Agrobiodiversity is broadly defined by the FAO as “the variety and variability of animals, plants and micro‐organisms that are used directly or indirectly for food and agriculture, including crops, livestock, forestry, and fisheries. It comprises the diversity of genetic resources (varieties, breeds) and species used for food, fodder, fibre, fuel, and pharmaceuticals. It also includes the diversity of non‐harvested species that support production (soil micro‐organisms, predators, pollinators), and those in the wider environment that support agro‐ecosystems (agricultural, pastoral, forest, and aquatic) as well as the diversity of the agro‐ecosystems” (FAO, 1999). Generally, increased agrobiodiversity leads to increased resilience to food system shocks and can dampen the negative effects of climate change on food production (Chivenge et al., 2015; Gonzalez, 2011; Inman et al., 2020; Ortiz, 2011). As an added benefit, agrobiodiversity is another protective barrier against the spread of crop diseases and buffers yield losses from biotic and abiotic stresses (Gonzalez, 2011). Increased agrobiodiversity also leads to better overall nutritional options, boosts local economies, and strengthens community independence and cultural preservation of native food systems (Bioversity International, 2020; Birol et al., 2006; Carney, 2021; Chatzopoulou et al., 2020; Dwivedi et al., 2013; Gonzalez, 2011; Mabhaudhi et al., 2019; Odhiambo et al., 2019; Tamariz, 2022). If incorporated appropriately, agrobiodiversity can also increase native biodiversity by emulating native environments (Gonzalez, 2011; Khumalo et al., 2012).

The most developed country in the region, South Africa, scores below average on Bioversity's International Agrobiodiversity Status Score (Avg. of 55%, South Africa = 52%), referencing to lack of emphasis on sustainable agricultural practices; lack of fruit, vegetable, seed, and nut consumption; and increased high‐sugar beverage consumption as key issues to public health and agriculture (Bioversity International, 2019). However, South Africa already has large integrated crop–livestock systems in addition to policies that protect indigenous genetic resource rights, which likely contributes to South Africa's Progress Score of 37%, which is slightly above the average of 32% (Bioversity International, 2019). Unfortunately, the other countries in the Southern African floristic regions do not currently have a calculated Agrobiodiversity Status Score to compare to that of South Africa. Integrated systems, policies, and incentives are important prerequisites for implementing the cultivation of native and indigenized crops. Namely, the presence of integrated agricultural systems and policy frameworks for genetic and intellectual property allow for faster development of new food systems.

Choosing suitable new crops to increase agrobiodiversity could be achieved by looking at traditional and indigenous crops rather than incorporating other “modern” crops available in other areas of the world. Both traditional and indigenous crops can be identified under the umbrella term of neglected and underutilized species (NUS), which are often more tolerant and adapted to local conditions than crops domesticated in other climates (Mabhaudhi, Chimonyo, & Modi, 2017). Therefore, these NUS would be more appropriate to use and domesticate in the area as NUS would be able to better adapt to Southern African climate conditions. However, these NUS will not be adopted and used without the development of clearly profitable value chains, incentive structures, and action plans (Mabhaudhi, Chimonyo, Chibarabada, & Modi, 2017). The first step towards this goal is to identify key NUS of interest that are well known, suited for the environment, and profitable for further research and development (Mabhaudhi, Chimonyo, Chibarabada, & Modi, 2017; Winstead et al., 2023).

Among South Africa's top 30 agricultural products in 2021, only sorghum (the 26th most produced agricultural product) is a native or indigenous crop variety to the region (Food and Agriculture Organization of the United Nations, 2019). The five topmost produced agricultural products in South Africa are sugar cane, maize, milk, potatoes, and wheat, which are all exotic to the area (Food and Agriculture Organization of the United Nations, 2019). This trend is similar for the other countries within the Southern African region with almost all production being from non‐native crops except for millet and sorghum (Food and Agriculture Organization of the United Nations, 2019).

About 62% of the Southern African floristic region's land is used for agriculture, yet only 5.05% of the total land area of the five countries is considered arable under conventional classifications, while the remainder of agricultural area is used predominantly as livestock grazing area (Bioversity International, 2019; The World Bank, 2023). Over 70% of land is used for agriculture in South Africa (960,000 km^2^), Eswatini (12,000 km^2^), and Lesotho (24,000 km^2^), while both Namibia (388,000 km^2^) and Botswana (258,000 km^2^) use about 46% of their land area for agriculture (Maxted & Vincent, 2021; The World Bank, 2023). However, it has been stated that conventional agricultural land use classifications do not take NUS into account, which is known to grow outside of “ideal” conditions and is more well suited to their native environments (Dubois et al., 2020; Mabhaudhi, Chimonyo, & Modi, 2017). This suggests that there is more arable land in Southern Africa than previously thought or classified.

A major limiting factor for agriculture in Southern Africa is water scarcity (Mabhaudhi, Chibarabada, & Modi, 2016). Therefore, water scarcity must be accounted for when proposing any changes or improvements to Southern African agriculture. This issue will likely get worse as Southern Africa is considered to be a climate change hotspot with temperatures increasing at a disproportionally higher rate than the rest of the world (Engelbrecht et al., 2024). A frequently proposed framework for viewing this problem holistically is through the water–energy–food nexus (WEF Nexus) framework, which focuses on the interconnectedness of these three resources. Many Southern African NUS require less water and have higher water use efficiency (WUE) than most current commercially grown crops in Southern Africa (Mabhaudhi, Chibarabada, & Modi, 2016). Additionally, many NUS are generally more nutrient dense than conventional crops (Mabhaudhi, Chimonyo, & Modi, 2017).

Although there are thousands of indigenous food and herbal medicinal plants found in Southern Africa, most are now only found in the wild. This includes leafy vegetables, indigenous fruits, grains, legumes, roots, tubers, and edible insects. The Department of Agriculture in South Africa carried out an inventory of the most common indigenous crops only listing 20, some of which are discussed below for drought tolerance (Department of Agriculture Forestry and Fisheries [DAFF], 2013). The introduction and overdependence on exotic species constitute a major impediment to the cultivation, distribution, diversity, abundance, and consumption of indigenous foods (Salami et al., 2022). Furthermore, the continued dependence on, and displacement of, traditional grains like sorghum and millet by maize has been cited as both environmentally and socially unsustainable (Fischer, 2022; Paumgarten et al., 2018). Most of the indigenous food sources are therefore little studied and mainly foraged and used for subsistence by communities and households in times of need when farmed crops or food in markets are not available (FAO et al., 2023). There is very little domestication of NUS, but in a few cases, these products have been nationally and internationally commercialized such as marula fruit (*Sclerocarya birrea*) produced for the popular beverage “amarula,” or sold in local markets such as mopane worm (*Gonimbrasia belina*) (Marshall et al., 2003; Neumann & Hirsch, 2000).

Prior knowledge and interest are key for the start of any domestication or cultivation efforts (Cerón‐Souza et al., 2021; Winstead et al., 2023). Therefore, this study aims to highlight which NUS in Southern Africa currently have the largest scientific knowledge base to encourage focused attention and that could be scaled up. To determine which NUS to focus on, we used the most recent and complete list of NUS in the Southern African floristic region compiled in 2019 (Welcome & van Wyk, 2019).

Importantly, long shelf lives and easy storage of food are important factors when building food resilience across seasons (Keding et al., 2013; Waldman et al., 2020). Current trends suggest that efficient food storage is a major contributor to agricultural resilience and economic stability (Bajželj et al., 2020). For Southern Africa, post‐harvest losses are quite high, so having naturally durable and storable agricultural products may decrease these losses (Dubois et al., 2020). For these reasons, we focused on Southern African NUS that are labeled as a “stored” food by the Welcome and van Wyk (2019) database. Although any food can be dried and stored long‐term using various modern methods, we focused on foods that were traditionally used as a “stored” food through traditional methods or because of the food's natural properties. Likewise, food that is easily stored provides a more resilient food source during or after disasters/catastrophe. This narrowing of focus also serves to narrow attention on only a few NUS rather than distribute interest across too many options and fail to stimulate further action.

From this list of stored NUS, we did a systematic literature review using Web of Science by Clarivate, to determine the relative scientific interest in these NUS. Likewise, NUS were further narrowed by additional criteria such as their purpose and domestication status. The hope of this study is to better direct researchers and policymakers to species of interest that already have large knowledgebases.

## 2 METHODS

The total number of plants listed as “stored” by Welcome and van Wyk (2019) was 80 plants out of 1740 wild edible plants (WEP). We excluded six stored wild edible plants because they were neither neglected nor underutilized given their status as a commodity by the FAO; cowpea (*Vigna unguiculata* (L.) Walp.), guava (*Psidium guajava* L.), common bean (*Phaseolus vulgaris* L.), date palm (*Phoenix dactylifera* L.), cassava (*Manihot esculenta* Crantz), and pumpkin (*Cucurbita pepo* L.). Among the remaining plants, exotic plants from Southern Africa were excluded (*n* = 16) and syrups, teas, “flavorants,” and beverages were excluded (*n* = 14) given their negligible nutritional value. This left 44 stored WEP we considered NUS to be reviewed using the above criteria.

Web of Science by Clarivate was used as the search database. The following search criteria were used with each of the 44 storable NUS: published, peer‐reviewed literature between the years 1982 and 2022, with the title containing the genus and species name of the plant. The research areas as defined by the Web of Science for every article in the literature search for each NUS were collected for each.

We further reduced the list of NUS in order to focus our attention on the plants that had the most scientific interest in their use as food. To do this we only included NUS that had any articles that were in the research areas of “food science and technology” and “nutrition and dietetics” (FaN articles) for each of the NUS. These research areas are assigned by the Web of Science Editors. These research areas are a “high‐pass filter,” and we recognize that there may be several papers related to food and nutrition that were not categorized as such. These categories helped us form a conservative conclusion on which species have relatively more scientific interest than others and do not serve as an objective measurement of interest. Each NUS's drought tolerance, status as a prioritized underutilized crop as described by Williams and Haq (2000), and ecological status were collected post hoc through literature review. The literature review for each of the NUS was not limited to the literature designated as a FaN article so there was no restriction on information gathered for each NUS. Please note that although the stored use of the NUS may be related to a specific plant part, the systematic literature review did not take this into account. However, this was taken into account when doing the final literature reviews on each of the 14 final species.

## 3 RESULTS AND DISCUSSION

**FIGURE 1:**
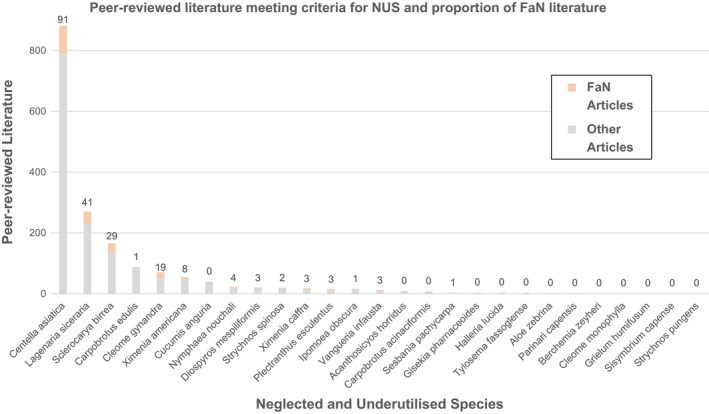
Graph showing total article counts for included NUS that had any articles meeting criteria. The number of FaN articles is displayed above each bar.

A total of 18,106 peer‐reviewed articles were found for all 80 stored NUS in the systematic literature search (Supplementary Material). However, this number drastically reduced when excluding FAO‐registered commodities (*n* = 13,250), non‐native NUS (*n* = 2717), and non‐nutritional foods (*n* = 357). The remaining 44 NUS had a combined 1730 peer‐reviewed articles among them (Figure 1). Many of the articles found referenced these NUS as being unwanted agricultural weeds rather than focusing on their cultivation. Only 14 of the NUS had any FaN articles (32%), showing that there is only a small group of storable, native, and nutritious NUS that have current interest among the scientific community (Table 1). Twenty of the final NUS had no articles containing their species name in the title in the last 40 years.

**TABLE 1:** List of 44 included NUS in systematic literature search.

Family	*Genus*	*Species*	Author	Common name	Part used	Stored use	Drought tolerant	Priority underutilized crop Williams and Haq (2000)	Ecological status	Total articles	Food Science & Technology	Nutrition & Dietetics	Drought tolerance source
Apeaceae	*Centella*	*asiatica*	(L.) Urb.	Gotu kola	Leaves	Vegetable	N	N	LC	881	65	26	Devkota and Kumar Jha (2011)
Cucurbitaceae	*Lagenaria*	*siceraria*	(Molina) Standl.	Calabash	Leaves, Fruit	Vegetable	Y	Y	N/A	270	30	11	Mashilo et al. (2017)
Anacardiaceae	*Sclerocarya*	*birrea*	(A.Rich.) Hochst.	Marula	Fruit	Snack	Y	Y	N/A	166	22	7	Muok and Ishii (2006)
Aizoaceae	*Carpobrotus*	*edulis*	(L.) L. Bolus	sour fig	Fruit	Snack	Y	N	N/A	88	1	0	Campoy et al. (2021)
Cleomaceae	*Cleome*	*gynandra*	L.	Shona cabbage	Leaves	Vegetable	N	N	N/A	71	12	7	Makaza et al. (2022)
Olacaceae	*Ximenia*	*americana*	L.	Hog plum	Fruit	Savory Preserve	Y	N	LC	55	5	3	Ekandjo (2015)
Cucurbitaceae	*Cucumis*	*anguria*	L.	Maroon cucumber	Leaves	Vegetable	N/A	Y	N/A	40	0	0	
Nymphaeaceae	*Nymphaea*	*nouchali*	Burm.f.	Blue lotus	Underground Storage Organ	Meal	N	N	LC	22	3	1	
Ebenaceae	*Diospyros*	*mespiliformis*	Hochst. ex A.DC.	Jackalberry	Fruit	Savory Preserve	Y	Y	LC	21	2	1	Olajuyigbe et al. (2012)
Loganiaceae	*Strychnos*	*spinosa*	Lam.	Natal orange	Fruit	Sweet Preserve	Y	Y	N/A	20	2	0	Nkosi et al. (2020)
Olacaceae	*Ximenia*	*caffra*	Sond.	Sourplum	Fruit	Sweet Preserve	Y	N	LC	18	2	1	Brennan (2008)
Lamiaceae	*Plectranthus*	*esculentus*	N.E.Br.	Livingstone potato	Underground Storage Organ	Vegetable	Y	Y	N/A	16	3	0	Allemann (2007)
Convolvulaceae	*Ipomoea*	*obscura*	(L.) Ker Gawl.	obscure morning glory	Leaves	Vegetable	N/A	N	N/A	16	1	0	
Rubiaceae	*Vangueria*	*infausta*	Burch.	Medlar	Fruit	Savory Preserve	Y	Y	LC	13	3	0	Karani et al. (2022)
Cucurbitaceae	*Acanthosicyos*	*horridus*	Welw. ex Hook.f.	Nara	Fruit	Sweet Preserve	Y	N	N/A	9	0	0	Hebeler (2000)
Aizoaceae	*Carpobrotus*	*acinaciformis*	(L.) L.Bolus	Elands sourfig	Fruit	Snack	Y	N	N/A	8	0	0	Huxley (1992)
Fabaceae	*Sesbania*	*pachycarpa*	DC.	Bãnfala dorodi	Flower	Snack	N/A	N	LC	4	1	0	
Stilbaceae	*Halleria*	*lucida*	L.	Tree fuchsiaa	Fruit	Snack	Y	N	LC	4	0	0	Palmer and Pitman (1972)
Fabaceae	*Tylosema*	*fassoglense*	(Schweinf.) Torre & Ebinger	Marama	Underground Storage Organ	Vegetable	Y	Y	N/A	4	0	0	Odhiambo (2020)
Gisekiaceae	*Gisekia*	*pharnaceoides*	L.	Gisekia	Leaves	Vegetable	N/A	N	N/A	4	0	0	
Chrysobalanaceae	*Parinari*	*capensis*	Harv.	Sand apple	Fruit	Preserve	N/A	N	N/A	2	0	0	
Asphodelaceae	*Aloe*	*zebrina*	Baker	Zebra leaf aloe	Flower	Vegetable	Y	N	LC	2	0	0	Cousins and Witkowski (2012)
Loganiaceae	*Strychnos*	*pungens*	Soler.	Spine‐leaved monkey orange	Fruit	Meal	Y	Y	LC	1	0	0	van Rayne et al. (2023)
Neuradaceae	*Grielum*	*humifusum*	Thunb.	NA	Underground Storage Organ	Meal	N/A	N	N/A	1	0	0	
Rhamnaceae	*Berchemia*	*zeyheri*	(Sond.) Grubov	Pink ivory	Fruit	Sweet Preserve	Y	N	LC	1	0	0	Dye et al. (2008)
Cleomaceae	*Cleome*	*monophylla*	L.	Ngélu	Leaves	Vegetable	N/A	N	N/A	1	0	0	
Brassicaceae	*Sisymbrium*	*capense*	Thunb.	Cape Mustard	Leaves	Vegetable	N/A	N	N/A	1	0	0	
Orchidaceae	*Eulophia*	*hereroensis*	Schltr.	NA	Stem	Meal	N/A	N	N/A	0	0	0	
Neuradaceae	*Grielum*	*grandiflorum*	(L.) Druce	Duikerwortel	Underground Storage Organ	Meal	N/A	N	N/A	0	0	0	
Iridaceae	*Psilosiphon*	*erythranthus*	(Klotzsch ex Klatt) Goldblatt & J.C.Manning	NA	Underground Storage Organ	Meal	N/A	N	N/A	0	0	0	
Tecophilaeaceae	*Walleria*	*nutans*	J.Kirk	NA	Underground Storage Organ	Meal	N/A	N	N/A	0	0	0	
Aizoaceae	*Carpobrotus*	*deliciosus*	(L.Bolus) L.Bolus	Delicious sour fig	Fruit	Snack	N/A	N	N/A	0	0	0	
Aizoaceae	*Carpobrotus*	*muirii*	(L.Bolus) L.Bolus	Dwarf sour fig	Fruit	Snack	N/A	N	N/A	0	0	0	
Aizoaceae	*Carpobrotus*	*quadrifidus*	L.Bolus	West Coast sour fig	Fruit	Snack	N/A	N	N/A	0	0	0	
Santalaceae	*Colpoon*	*compressum*	P.J.Bergius	Cape sumach	Fruit	Snack	N/A	N	N/A	0	0	0	
Malvaceae	*Grewia*	*avellana*	Hiern	Wild Jute	Fruit	Snack	N/A	N	N/A	0	0	0	
Anacardiaceae	*Searsia*	*burchellii*	(Sond. Ex Engl.) Moffett	Karoo kunibush	Fruit	Snack	N/A	N	LC	0	0	0	
Anacardiaceae	*Searsia*	*laevigata*	(L.) F.A.Barkley	Dune currant rhus	Fruit	Snack	N/A	N	LC	0	0	0	
Asphodelaceae	*Aloe*	*esculenta*	L.C.Leach	NA	Flower	Vegetable	Y	N	N/A	0	0	0	Cousins and Witkowski (2012)
Aizoaceae	*Aizoon*	*glinoides*	L.f.	Ucwethekazi	Leaves	Vegetable	N/A	N	N/A	0	0	0	
Cucurbitaceae	*Coccinia*	*rehmannii*	Cogn.	Wild cucumber	Underground Storage Organ	Vegetable	N/A	N	N/A	0	0	0	
Cyperaceae	*Cyperus*	*fulgens*	C.B.Clarke	NA	Underground Storage Organ	Vegetable	N/A	N	N/A	0	0	0	
Amaranthaceae	*Hermbstaedtia*	*argenteiformis*	Schinz	NA	Leaves	Vegetable	N/A	N	N/A	0	0	0	
Brassicaceae	*Lepidium*	*schinzii*	Thell.	Schinz's pepperweed	Leaves	Vegetable	N/A	N	N/A	0	0	0	

Out of the final list of 44, we found 20 NUS with drought‐tolerance information available, 85% of which were considered to be drought tolerant. This bolsters previous arguments that using more NUS would result in more drought‐resilient agricultural systems. The combination of drought resilience and long shelf life should make these NUS extremely desirable to potential agricultural developers and consumers. Additionally, we have found that many of these NUS are rich in vitamins and minerals as we highlight below. The authors recognize a bias in results as only English scientific literature was searched. However, this should not be a huge issue as English is the lingua franca for the scientific community in Southern Africa. Although there are surely other sources of information in other languages available, our pursuit is only related to scientific interest. Therefore, this bias theoretically should not skew our results toward or against any NUS.

### 3.1 Literature overviews of 14 NUS with FaN articles

#### 3.1.1 Centella asiatica

Also known as Asiatic pennywort and gotu kola, this is a small forb native to Southern Africa. The leaves have high levels of vitamin A and iron and have been recommended for introduction into diets with vitamin deficiencies (Mertz et al., 2019). Past work on how to process leaves and petioles in relation to their flavor profile has also already been studied (Wongfhun et al., 2010). Others have similarly tested sensory perception and nutrition of juices made from the leaves with success in producing positive results among taste testers (Junsi & Siripongvutikorn, 2022). Additionally, *C. asiatica* is shade tolerant, which could be important for a resilient GCR crop (Priyanka et al., 2022).

#### 3.1.2 Lagenaria siceraria

This species is native to several continents and has been cultivated at small scales throughout human history across the globe (Erickson et al., 2005). Although there is a risk of poisoning if eating bitter fruits (Ho et al., 2014), the seeds are high in fat, protein, and minerals (Ogunbusola, 2018). Rind wastes from *L. siceraria* fruit are a source of cellulose nanocrystals, which have many uses including several pharmaceutical applications (George & Sabapathi, 2015; Meda et al., 2022). It can also be used as a fiber additive in processed foods (Verma et al., 2012). A prior study has shown that the clarified juice of *L. siceraria* fruit has a shelf life and taste that are appealing to potential buyers (Mondal et al., 2016). Overall, given global familiarity with calabash and its multiple uses, it is a good option for investing in its further development and commercialization.

#### 3.1.3 Sclerocarya birrea

This fruit, commonly referred to as Marula is a well‐known commercialized product in rural Southern Africa, and although there is a large genetic and geographical stock, there is a large degree of phenotypic variation among populations (Nyoka et al., 2015). Fruits and seeds are high in vitamin C, antioxidants, protein, fat, magnesium, phosphorus, and potassium (Hiwilepo‐van Hal et al., 2014). It has been intercropped in fields for centuries and is commonly used to make fermented drinks, is an important part of rural economies, and is one of the most widely used NUS in sub‐Saharan Africa (Hiwilepo‐van Hal et al., 2013). This species is also predicted to do well and even expand its range at the current rate of climate change (Jinga et al., 2022). Most parts of the plant have some sort of practical use including cosmetics, pharmaceuticals, seasoning, compost, animal feed, meat preservative, etc. (Mashau et al., 2022). In addition to its popular use in making marula beer and Amarula liqueur, marula nut is high in protein and is a viable source of plant‐based protein (Malebana et al., 2018).

#### 3.1.4 Cleome gynandra

Several recent studies suggest that *C. gynandra* could be one of the most viable and nutritious NUS in Southern Africa right now (Omondi et al., 2017; van den Heever & Venter, 2007). It is an important leafy vegetable native to South Africa and is known for being an important staple during the relish‐gap period and leaves are used as a potherb, relish, stew, or side (van den Heever & Venter, 2007). The leaves are dried and mixed with other foods, and the leaves are known to be high in vitamins A, C, and E, calcium, and iron, although vitamin content drastically reduces after traditional drying methods (Moyo & Aremu, 2022; van den Heever & Venter, 2007). Work on collecting gene accessions and determining current morphology types has already shown that it has a wide genetic variability and great potential as a resilient and versatile crop (Omondi et al., 2017). Additionally, the ethanolic extract of *C. gynandra* leaves has been shown to have anti‐inflammatory effects, as well as a vast array of unstudied biologically active compounds (Maina et al., 2021; Narendhirakannan et al., 2007).

#### 3.1.5 Ximenia americana

Most of the literature relating to the nutritional properties of *X. americana* relate to its bioactive constituents such as antioxidants and vitamin C content (Almeida et al., 2016; Darcio et al., 2015). Studies have also shown that the leaves of *X. americana* are very high in calcium, vitamin C, and essential fatty acids (Freiberger et al., 1998; Galdino et al., 2008).

#### 3.1.6 Nymphaea nouchali

Literature suggests that *N. nouchali* rhizomes are healthy antioxidant‐rich food for those with hypoglycemia (Alam et al., 2021; Anand et al., 2021). A study found that *N. nouchali* flowers are also a good source of many nutraceuticals and minerals (Dias et al., 2021). Vitamins and minerals found to be in *N. nouchali* tubers are ascorbic acid, niacin, riboflavin, thiamin, calcium, iron, and zinc (Anand et al., 2019).

#### 3.1.7 Diospyros mespiliformis

Nutritional analysis of the seeds of *D. mespiliformis* shows that they are a rich source of carbohydrates and omega‐3 fatty acids (Chivandi & Erlwanger, 2011). The fruits of *D. mespiliformis* have also been found to have antioxidant effects and benefits of nutraceutical applications (Ndhlala, Chitindingu, et al., 2008). The stored use for *D. mespiliformes* is in preserved fruit, which provides meaningful contributions to zinc, iron, vitamin A, and beta‐carotene to the diet, and its dry matter content is 10% protein (Achaglinkame et al., 2019). Additionally, the roots, leaves, bark, and stems of *D. mespiliformis* are regularly used medicinally which adds to its market value (Ramadwa & Meddows‐Taylor, 2023).

#### 3.1.8 Vangueria infausta

The acidic fruits of *V. infausta* can be cooked and dried with sugar to create a “fruit leather” with water activity ≤0.6, which is low enough to stifle the growth of microorganisms and allows for the safe long‐term storage of this fruit (Khan et al., 2020). Drying the fruit for later use is documented as a traditional storage method, as well as having a high ash content (Magaia et al., 2013). There was no literature found that related to its nutritional value.

#### 3.1.9 Ximenia caffra

Studies have shown that the fruits of *X. caffra* are a significant source of vitamin E, calcium, copper, magnesium, phosphorus, and zinc (Lekoba et al., 2024). Several papers reference the fruits' polyphenolic content mostly consisting of flavonoids and their potential as antioxidants (Ndhlala, Muchuweti, et al., 2008; Oosthuizen et al., 2018). The fruit kernels have also been shown to be high in many important saturated fatty acids as well as nervonic acid (Chivandi et al., 2008).

#### 3.1.10 Plectranthus esculentus

Tubers of this plant are known to be a cheap source of carbohydrates, containing 854 g/kg carbohydrates DW; however, studies also show that they may have a high lead content (Temple et al., 1991). South African varieties of *P. esculentus* are significantly higher in micronutrients and protein than other common vegetables; namely high in vitamin A (0.17 mg/100 g DM), iron (50.4 mg/100 g DM), calcium (140.3 mg/100 g DM), and protein (13.5 g/100 g DM), which are essential in malnourished and disaster‐prone areas (Allemann & Hammes, 2003). They also have a moderate glycemic index ranging from 55 to 70 depending on the cooking method and do have several flavonoid antioxidants present (Eleazu et al., 2016; Eleazu & Eleazu, 2015). A study by Ezeocha and Ironkwe (2017) found that there are several effective ways of easily storing *P. esculentus* tubers underground and retaining nutritional value by covering it with wood shavings, sand, or ash. Additionally, they also found that storing the tubers by simply burying them underground resulted in minimal rot or sprouting (Ezeocha & Ironkwe, 2017).

#### 3.1.11 Strychnos spinosa

A famine food that can be rich in antioxidants (Nhukarume et al., 2010). Fruits have mineral values that are comparable to other major fruits, and have great variation in fruit characteristics, lending itself to possibilities of many cultivation variety development (Sitrit et al., 2003). The fruits of *S. spinosa* are very high in fat (31.3 g /kg DW), which contributes to their very high energy value of 1923 kJ/100 g (Saka & Msonthi, 1994). However, more recent studies do not have fat contents nearly this high (Mbhele et al., 2024). A population with favorable domestication traits such as fruit mass and tree diameter has already been identified in Sudano‐Guinean zone of Benin (Avakoudjo et al., 2021). There have also been studies on the nutritional and genetic properties of different morphotypes of *S. spinosa* in KwaZulu‐Natal, South Africa (Mbhele et al., 2023, 2024). There seems to be growing interest in *S. spinosa* as an economically profitable crop if postharvest processing improves given its popularity, nutritional value, and suitability to the Southern African climate (Omotayo & Aremu, 2021).

#### 3.1.12 Carpobrotus edulis

This is a halophytic plant that grows well in arid, coastal climates. Its fruits are a good source of fiber as well as carbohydrates, Ca, Na, Mn, Zn, Fe, and polyunsaturated fatty acids (Neves et al., 2021; Pereira et al., 2023). Although only the fruit is listed as stored food, the leaves and flowers of *C. edulis* are also edible. Similar to *S. spinosa*, studies measured very wide ranges of protein contents for *C. edulis* fruits from 23.5 g /100 g DM to 3.45 g/ 100 g DM (Sibiya et al., 2021).

#### 3.1.13 Sesbania pachycarpa

One study on *S. pachycarpa* focused on its nutritional value as a possible ruminant feed. However, this paper did report a high protein and fiber content of leaf dry mass (237 g/kg DM and 321 g/kg DM, respectively) (Muetzel et al., 2003). Likewise, the seeds have been studied for their fat content in the past and not their flowers as described in van Wyk database (Glew et al., 2005).

#### 3.1.14 Ipomoea obscura

The one citation for *I. obscura* that was in the Food Science and Technology research area category was focused on possible antiviral chemical constituents of *I. obscura* (Poochi et al., 2020). This reference likely should not have been put into the category of Food Science and Technology or our final list, but regardless, it has been included in this review. No other papers about its nutritional value or food value were found.

### 3.2 Most promising NUS prospects

After reviewing the literature for all of the NUS with FaN articles, there are a few that stand out as NUS that have much more of a scientific knowledge base and development than others. Some NUS already have had genetic and physiological studies to determine potential cultivars and germplasm (*S. birrea*, *Cleome gynandra*, and *Strychnos spinosa*). Many of the NUS have already had nutritional analysis including proximate analysis and micronutrient analyses. In particular, *C. gynandra* has a rich scientific knowledge base, high nutritional quality, cultural importance, and a general scientific consensus that it may have the most potential to be profitable and nutritionally important. Also noteworthy is the subsistence potential of *Plectranthus esculentus* tubers as they are known to be cheap, reliable, nutritious, and easily stored just by burying them. In terms of unique NUS that could be profitable for marginal markets, *Carpobrotus edulis* could make use of arid and salty soil that would otherwise be unusable for agriculture. Additionally, *C. edulis* has multiple uses for multiple parts of the plant and a wide phenotypic range giving it a broad economic potential to fill multiple producer and consumer niches.

### 3.3 Storage and drying methods

Several studies show the importance of both the drying method and storage method of foods in the retention of their nutritional value and quality. Leaves can retain significant nutritional value when dried, especially if dried in the shade, or by using a microwave. Although it is true that drying reduces nutritional value, it also increases nutritional density. Therefore, the prospect of using leaf vegetables as nutritious stored food is still of great value (Babu et al., 2018).

The most important traditional methods for preserving foods in Africa are lactic acid fermentation and air‐/sun‐drying (Aworh, 2023). These methods are passed on through generations and allow for the transition of some plants from inedible to edible. These techniques are also low cost and do not require electricity. Proper storage of crops after the drying process is equally important. Hermetic storage (air‐tight) is one of the most reliable forms of storage and has been an important alternative for decreasing post‐harvest losses. The most notable of these storage methods are vacuum‐sealed bags, hermetic metal silos, and metal drums (Sikora et al., 2020). These storage methods are simple and require little to no electricity for maintenance.

### 3.4 Economic value of NUS

There are mature formal markets for most of the major staple foods and crops in Southern Africa. Introducing NUS will require building new local and regional markets. Because NUS have not been mainstreamed, they have potential prospects of generating new jobs and income opportunities, especially for communities that have knowledge of the wild crops which include rural small‐hold farmers and women. Promoting NUS can integrate the informal sector which usually exists for NUS since developed markets are absent (Piñeiro et al., 2020). New markets will require public incentives and investments across the supply chain in infrastructure development, regulations, and subsidies for actors along the supply chain, along with promotion and consumer awareness on the demand side. Not only will NUS promotion help create alternative income and job opportunities but it can also build resilience to climate change by diversifying agriculture and food systems. Adding variety to diets will also enhance nutrition to help address Africa's malnutrition and poverty problems (Hunter et al., 2019). Since many NUS are more drought tolerant than the major staple crops, they will require less water and possibly less fertilizers and pesticides, implying less input production costs. Relying less on major staple crops can improve sustainable land management while enhancing profitability and community empowerment.

The lack of a large market consumer base is a well‐known issue for mainstreaming NUS (van Zonneveld et al., 2023). Although introducing these NUS to the mainstream market would likely not attract much support given the current lack of profit and perceived market demand, NUS could serve as an important and intentionally disruptive innovation. Disruptive innovations start small and appeal to a small set of fringe consumers (and producers), yet provide functionality that mainstream products do not offer. This allows the innovation to start developing a consumer base and grow in popularity and functionality, sometimes surpassing the original product (Christensen et al., 2018). This economic theory could apply to the development of new crops and provide a basis for the initial development of NUS crops (Khan & Arif, 2023). Each of these have unique attributes and abilities not offered by current mainstream crops such as significant drought resilience, higher micronutrient density, cultural importance, and flavor (Curry et al., 2021). If incentivized to a small group of producers aimed at small, specific groups of consumers, the likelihood of success in developing NUS cultivation could increase drastically.

### 3.5 Next steps

Many publications have now been written on the prioritization of crops, but there still is a lack of actionable next steps to progress. Here, we have collected and discussed NUS that have major potential for further development and profitability. Next steps should include the calculation and evaluation of readiness and use scores of the most highly researched NUS from this study (Sartas et al., 2020). The calculation of these scores for each step in cultivation is a time‐intensive process for each crop and requires input from experts of the crop of interest within the region to determine which steps are most important. The results from this scoring method on the top studied NUS should be used by partnering with local entities to champion the development of NUS. Perhaps a faster initial option would be to use the scoring technique proposed by Thornton et al. (2024), which follows the assumption of Sartas et al. (2020) that any innovation package is only as strong as its weakest innovation/sector. Thornton et al. (2024) creates clear categories to be scored that are relevant to crop development and uses golden rice as an example of this principle. Although there is sufficient technological development and supply of golden rice, there is no “social license” to actually grow golden rice and therefore there is no demand or willingness to grow it.

NUS that have been highlighted in this paper have already shown to have scientific interest, nutritional value, and drought resilience meeting prerequisites for further development. The goal is for this paper to serve as a springboard for local organizations to justify the funding of these needed next steps. Our paper also may serve as a template for future studies in other areas of the world that also require the identification of profitable NUS for development.

As described by Mabhaudi et al. (2017), most research and discussion about NUS has remained theoretical with no standardized way of applying the growing knowledge base and development of Southern African NUS. One important step in their proposed roadmap was the identification of NUS of interest that can be championed by NGOs and other organizations to bring this knowledge to organizations and government agencies that can turn theoretical work into applications. These strategies could include the incorporation of NUS into current Food Systems Resilience Program for Eastern and Southern Africa (FSRP), possibly through the cooperation with The Centre for Coordination of Agricultural Research and Development for Southern Africa (CCARDESA).

## 4 CONCLUSION

In this systematic review, we have shown that there are several NUS that are suited for growth in Southern Africa that have a knowledge base, nutritional value, and agricultural interest that warrants their further study and development by local entities. We highly recommend that the domestication and scaling up of the highlighted NUS be invested in for the improvement of Southern African agrobiodiversity and food resilience by local groups; namely *S. birrea*, *C. gynandra*, *S. spinosa*, *P. esculentus*, and *C. edulis*. We recommend that producers of these new crops focus on fringe market consumer groups to create demand and improve development of the NUS. Not only are these NUS nutritious but they can also be stored easily throughout the year, providing longer and more stable shipping opportunities and improved nutritional and economic resilience through disasters, catastrophes, or climate change.

## CONFLICT OF INTEREST STATEMENT

The authors declare no competing interests.

## Supporting information


Data S1.

## Data Availability

Data for systematic search are available in the Supplementary Material.

## References

[pei370004-bib-0001] Achaglinkame, M. A. , Aderibigbe, R. O. , Hensel, O. , Sturm, B. , & Korese, J. K. (2019). Nutritional characteristics of four underutilized edible wild fruits of dietary interest in Ghana. Food, 8(3), 104. 10.3390/foods8030104 PMC6463063 30897690

[pei370004-bib-0002] Alam, M. B. , Naznin, M. , Islam, S. , Alshammari, F. H. , Choi, H. J. , Song, B. R. , Kim, S. , & Lee, S. H. (2021). High resolution mass spectroscopy‐based secondary metabolite profiling of *Nymphaea nouchali* (Burm. f) stem attenuates oxidative stress via regulation of MAPK/Nrf2/HO1/ROS pathway. Antioxidants, 10(5), 719. 10.3390/antiox10050719 34063678 PMC8147620

[pei370004-bib-0003] Allemann, J. (2007). *Literature review of the Livingstone potato (Plectranthus esculentus N.E.Br.)* [PhD thesis]. University of Pretoria.

[pei370004-bib-0004] Allemann, J. , & Hammes, P. S. (2003). Chemical composition of south African *Plectranthus esculentus* tubers. South African Journal of Science, 99, 127–129.

[pei370004-bib-0005] Almeida, M. L. B. , Freitas, W. E. D. S. , de Morais, P. L. D. , Sarmento, J. D. A. , & Alves, R. E. (2016). Bioactive compounds and antioxidant potential fruit of *Ximenia americana* L. Food Chemistry, 192, 1078–1082. 10.1016/j.foodchem.2015.07.129 26304450

[pei370004-bib-0006] Anand, A. , Komati, A. , Katragunta, K. , Shaik, H. , Nagendla, N. K. , Kuncha, M. , Mudiam, M. K. R. , Babu, K. S. , & Tiwari, A. K. (2021). Phytometabolomic analysis of boiled rhizome of *Nymphaea nouchali* (Burm. f.) using UPLC‐Q‐TOF‐MS^E^, LC‐QqQ‐MS & GC–MS and evaluation of antihyperglycemic and antioxidant activities. Food Chemistry, 342, 128313. 10.1016/j.foodchem.2020.128313 33067043

[pei370004-bib-0007] Anand, A. , Priyanka, U. , Lakshma Nayak, V. , Zehra, A. , Suresh Babu, K. , & Tiwari, A. K. (2019). Nutritional composition and antioxidative stress properties in boiled tuberous rhizome of Neel Kamal (*Nymphaea nouchali* Burm. f.). Indian Journal of Natural Products and Resources, 10(1), 59–67.

[pei370004-bib-0008] Avakoudjo, H. G. G. , Idohou, R. , Salako, K. V. , Hounkpèvi, A. , Koné, M. W. , & Assogbadjo, A. E. (2021). Diversity in tree and fruit traits of *Strychnos spinosa* Lam. along a climatic gradient in Benin: A step towards domestication. Genetic Resources and Crop Evolution, 68(6), 2423–2440. 10.1007/s10722-021-01140-5

[pei370004-bib-0009] Aworh, O. C. (2023). African traditional foods and sustainable food security. Food Control, 145, 109393. 10.1016/j.foodcont.2022.109393

[pei370004-bib-0010] Babu, A. K. , Kumaresan, G. , Raj, V. A. A. , & Velraj, R. (2018). Review of leaf drying: Mechanism and influencing parameters, drying methods, nutrient preservation, and mathematical models. Renewable and Sustainable Energy Reviews, 90, 536–556. 10.1016/j.rser.2018.04.002

[pei370004-bib-0011] Bajželj, B. , Quested, T. E. , Röös, E. , & Swannell, R. P. J. (2020). The role of reducing food waste for resilient food systems. Ecosystem Services, 45, 101140. 10.1016/j.ecoser.2020.101140 32834962 PMC7392851

[pei370004-bib-0012] Barrett, A. M. , Baum, S. D. , & Hostetler, K. (2013). Analyzing and reducing the risks of inadvertent nuclear war between the United States and Russia. Science and Global Security, 21, 106–133. 10.1080/08929882.2013.798984

[pei370004-bib-0013] Bioversity International . (2019). Agrobiodiversity index report 2019: Risk and resilience . https://hdl.handle.net/10568/100820

[pei370004-bib-0014] Bioversity International . (2020). Agrobiodiversity, school gardens and healthy diets: Promoting biodiversity, food and sustainable nutrition (D. Hunter, E. Monville‐Oro, B. Burgos, C. N. Rogel, B. Calub, J. Gonsalves, & N. Lauridsen, Eds.). Routledge.

[pei370004-bib-0015] Birol, E. , Smale, M. , & Gyovai, A. (2006). Using a choice experiment to estimate farmers' valuation of agrobiodiversity on Hungarian small farms. Environmental and Resource Economics, 34, 439–469. 10.1007/s10640-006-0009-9

[pei370004-bib-0016] Brennan, R. (2008). The encyclopedia of fruit & nuts (J. Janick & R. E. Paull, Eds.). CABI Publishing.

[pei370004-bib-0017] Campoy, J. G. , Lema, M. , Fenollosa, E. , Munné‐Bosch, S. , & Retuerto, R. (2021). Functional responses to climate change may increase invasive potential of *Carpobrotus edulis* . American Journal of Botany, 108(10), 1902–1916. 10.1002/ajb2.1745 34636413

[pei370004-bib-0018] Carney, J. A. (2021). Subsistence in the Plantationocene: Dooryard gardens, agrobiodiversity, and the subaltern economies of slavery. Journal of Peasant Studies, 48(5), 1075–1099. 10.1080/03066150.2020.1725488

[pei370004-bib-0019] Cerón‐Souza, I. , Galeano, C. H. , Tehelen, K. , Jiménez, H. R. , & González, C. (2021). Opportunities and challenges to improve a public research program in plant breeding and enhance underutilized plant genetic resources in the tropics. Genes, 12, 1584. 10.3390/genes12101584 34680981 PMC8535561

[pei370004-bib-0020] Chatzopoulou, E. , Carocho, M. , Di Gioia, F. , & Petropoulos, S. A. (2020). The beneficial health effects of vegetables and wild edible greens: The case of the Mediterranean diet and its sustainability. Applied Sciences (Switzerland), 10(24), 1–27. 10.3390/app10249144

[pei370004-bib-0021] Chivandi, E. , Davidson, B. C. , & Erlwanger, K. H. (2008). A comparison of the lipid and fatty acid profiles from the kernels of the fruit (nuts) of *Ximenia caffra* and *Ricinodendron rautanenii* from Zimbabwe. Industrial Crops and Products, 27(1), 29–32. 10.1016/j.indcrop.2007.06.002

[pei370004-bib-0022] Chivandi, E. , & Erlwanger, K. H. (2011). Potential usage of African ebony (*Diospyros mespiliformis*) seeds in human health. In V. R. Preedy , R. R. Watson , & V. B. Patel (Eds.), Nuts and seeds in health and disease prevention (pp. 147–152). Elsevier.

[pei370004-bib-0023] Chivenge, P. , Mabhaudhi, T. , Modi, A. T. , & Mafongoya, P. (2015). The potential role of neglected and underutilised crop species as future crops under water scarce conditions in sub‐Saharan Africa. International Journal of Environmental Research and Public Health, 12(6), 5685–5711. 10.3390/ijerph120605685 26016431 PMC4483666

[pei370004-bib-0024] Christensen, C. M. , McDonald, R. , Altman, E. J. , & Palmer, J. E. (2018). Disruptive innovation: An intellectual history and directions for future research. Journal of Management Studies, 55(7), 1043–1078. 10.1111/joms.12349

[pei370004-bib-0025] Coupe, J. , Bardeen, C. G. , Robock, A. , & Toon, O. B. (2019). Nuclear winter responses to nuclear war between the United States and Russia in the whole atmosphere community climate model version 4 and the Goddard Institute for Space Studies ModelE. Journal of Geophysical Research: Atmospheres, 124(15), 8522–8543. 10.1029/2019JD030509

[pei370004-bib-0026] Cousins, S. R. , & Witkowski, E. T. F. (2012). African aloe ecology: A review. Journal of Arid Environments, 85, 1–17. 10.1016/j.jaridenv.2012.03.022

[pei370004-bib-0027] Curry, G. N. , Nake, S. , Koczberski, G. , Oswald, M. , Rafflegeau, S. , Lummani, J. , Peter, E. , & Nailina, R. (2021). Disruptive innovation in agriculture: Socio‐cultural factors in technology adoption in the developing world. Journal of Rural Studies, 88, 422–431. 10.1016/j.jrurstud.2021.07.022

[pei370004-bib-0028] Darcio, J. D. A. , de Morais, P. L. D. , de Souza, F. I. , da Costa, L. R. , & de Assis Melo, N. J. (2015). Bioactive compounds and antioxidant activity of *Ximenia americana* coming from different collection sites. Archivos Latinoamericanos de Nutrición, 65(4), 263–270.

[pei370004-bib-0029] de Waal, A. , & Whiteside, A. (2003). New variant famine: AIDS and food crisis in southern Africa. Lancet, 362(9391), 1234–1237. 10.1016/S0140-6736(03)14548-5 14568749

[pei370004-bib-0030] Denkenberger, D. , & Pearce, J. (2015). Feeding everyone: Solving the food crisis in event of global catastrophes that kill crops or obscure the sun. Futures, 72, 57–68. 10.1016/j.futures.2014.11.008

[pei370004-bib-0031] Department of Agriculture Forestry and Fisheries (DAFF) . (2013). Most common indigenous food crops of South Africa .

[pei370004-bib-0032] Devkota, A. , & Kumar Jha, P. (2011). Influence of water stress on growth and yield of *Centella asiatica* . International Agrophysics, 25(3), 211–214.

[pei370004-bib-0033] Dias, O. , Tungare, K. , Palamthodi, S. , & Bhori, M. (2021). *Nymphaea nouchali* Burm. F. flowers as a potential food additiveand revitalizer: A biochemico‐toxicological insight. Journal of Food Processing and Preservation, 45(5), e15405. 10.1111/jfpp.15405

[pei370004-bib-0034] Dubois, T. , Nordey, T. , & Opara, U. L. (2020). Unleashing the power of vegetables and fruits in Southern Africa. In R. A. Sikora , E. R. Terry , P. L. G. Vlek , & J. Chitja (Eds.), Transforming agriculture in southern Africa: Constraints, technologies, policies and processes (pp. 170–178). Taylor & Francis.

[pei370004-bib-0035] Dwivedi, S. , Sahrawat, K. , Upadhyaya, H. , & Ortiz, R. (2013). Food, nutrition and agrobiodiversity under global climate change. Advances in agronomy, 120, 1–128. 10.1016/B978-0-12-407686-0.00001-4

[pei370004-bib-0036] Dye, P. J. , Gush, M. B. , Everson, C. S. , Jarmain, C. , Clulow, A. , Mengistu, M. , Geldenhuys, C. J. , Wise, R. , Scholes, R. J. , Archibald, S. , & Savage, M. J. (2008). Water use in relation to biomass of indigenous tree species in woodland, forest and/or plantation conditions . Water Research Commission Report (361/08).

[pei370004-bib-0037] Ekandjo, A. (2015). *Genetic analysis of* Ximenia Americana *under natural conditions* [Master of Science]. University of Namibia.

[pei370004-bib-0038] Eleazu, C. O. , & Eleazu, K. C. (2015). Nutrient composition, antioxidant capacity and natural products in Livingstone potato (*Plectranthus esculentus*). Journal of Food Processing and Preservation, 39(6), 3050–3058. 10.1111/jfpp.12570

[pei370004-bib-0039] Eleazu, C. O. , Eleazu, K. C. , & Iroaganachi, M. (2016). In vitro starch digestibility, α‐amylase and α‐glucosidase inhibitory capacities of raw and processed forms of three varieties of Livingstone potato (*Plectranthus esculentus*). Innovative Food Science and Emerging Technologies, 37, 37–43. 10.1016/j.ifset.2016.08.007

[pei370004-bib-0040] Engelbrecht, F. A. , Steinkopf, J. , Padavatan, J. , & Midgley, G. F. (2024). Projections of future climate change in Southern Africa and the potential for regional tipping points. In G. P. von Maltitz , G. F. Midgley , J. Veitch , C. Brümmer , R. P. Rötter , F. A. Viehberg , & M. Veste (Eds.), Sustainability of southern African ecosystems under global change (pp. 169–190). Springer International Publishing.

[pei370004-bib-0041] Erickson, D. L. , Smith, B. D. , Clarke, A. C. , Sandweiss, D. H. , & Tuross, N. (2005). An Asian origin for a 10,000‐year‐old domesticated plant in the Americas. PNAS, 102(51), 18315–18320. 16352716 10.1073/pnas.0509279102 PMC1311910

[pei370004-bib-0042] Ezeocha, C. V. , & Ironkwe, A. G. (2017). Effect of storage methods and period on the physiological and nutrient components of livingstone potato (*Plectranthus esculentus*) in Abia State, Nigeria. Open Agriculture, 2(1), 213–219. 10.1515/opag-2017-0022

[pei370004-bib-0043] FAO . (1999). Agricultural biodiversity. *FAO multifunctional character of agriculture and land: Conference background paper no. 1*.

[pei370004-bib-0044] FAO , IFAD , UNICEF , WFP , & WHO . (2023). The state of food security and nutrition in the world 2023. In The state of food security and nutrition in the world 2023. FAO. 10.4060/cc3017en

[pei370004-bib-0045] Fischer, K. (2022). Why Africa's new green revolution is failing—Maize as a commodity and anti‐commodity in South Africa. Geoforum, 130, 96–104. 10.1016/j.geoforum.2021.08.001

[pei370004-bib-0046] Food and Agriculture Organization of the United Nations . (2019). FAOSTAT statistical database. FAO.

[pei370004-bib-0047] Freiberger, C. E. , Vanderjagt, D. J. , Pastuszyn, A. , Glew, R. S. , Mounkaila, G. , Millson, M. , & Glew, R. H. (1998). Nutrient content of the edible leaves of seven wild plants from Niger. Plant Foods for Human Nutrition, 53, 57–69. 10890758 10.1023/a:1008080508028

[pei370004-bib-0048] Galdino, G. , Silva, D. A. , de Souza, A. , Lígia, P. , de Morais, D. , Cabral, E. , Santos, D. , Moura, R. D. , & Barbosa Menezes, J. (2008). Caracterização do fruto de ameixa silvestre (*Ximenia americana*). Revista Brasileira de Fruticultura, 30(2), 311–314.

[pei370004-bib-0049] Garner, A. J. , Mann, M. E. , Emanuel, K. A. , Kopp, R. E. , Lin, N. , Alley, R. B. , Horton, B. P. , Deconto, R. M. , Donnelly, J. P. , & Pollard, D. (2017). Impact of climate change on New York City's coastal flood hazard: Increasing flood heights from the preindustrial to 2300 CE. The Proceedings of the National Academy of Sciences, 114(45), 11861–11866. 10.1073/pnas.1703568114 PMC5692530 29078274

[pei370004-bib-0050] George, J. , & Sabapathi, S. N. (2015). Cellulose nanocrystals: Synthesis, functional properties, and applications. Nanotechnology, Science and Applications, 8, 45–54. 10.2147/NSA.S64386 26604715 PMC4639556

[pei370004-bib-0051] Glew, R. S. , VanderJagt, D. J. , Bosse, R. , Huang, Y. S. , Chuang, L. T. , & Glew, R. H. (2005). The nutrient content of three edible plants of the Republic of Niger. Journal of Food Composition and Analysis, 18(1), 15–27. 10.1016/j.jfca.2003.12.002

[pei370004-bib-0052] Gonzalez, C. G. (2011). Climate change, food security, and agrobiodiversity: Toward a just, resilient, and sustainable food system. Fordham Environmental Law Review, 22(3), 493–522.

[pei370004-bib-0053] Hebeler, F. (2000). Structural and ecophysiological shoot features of the leafless cucurbit Acanthosicyos horridus, a keystone endemic of the Namib desert. Justus‐Liebig‐Universität Giessen.

[pei370004-bib-0054] Hiwilepo‐van Hal, P. , Bille, P. G. , Verkerk, R. , & Dekker, M. (2013). The effect of temperature and time on the quality of naturally fermented marula (*Sclerocarya birrea* subsp. Caffra) juice. LWT‐Food Science and Technology, 53(1), 70–75. 10.1016/j.lwt.2013.02.021

[pei370004-bib-0055] Hiwilepo‐van Hal, P. , Bille, P. G. , Verkerk, R. , van Boekel, M. A. J. S. , & Dekker, M. (2014). A review of the proximate composition and nutritional value of Marula (*Sclerocarya birrea* subsp. *caffra*). Phytochemistry Reviews, 13(4), 881–892. 10.1007/s11101-014-9352-6

[pei370004-bib-0056] Ho, C. H. , Ho, M. G. , Ho, S. P. , & Ho, H. H. (2014). Bitter bottle gourd (*Lagenaria siceraria*) toxicity. Journal of Emergency Medicine, 46(6), 772–775. 10.1016/j.jemermed.2013.08.106 24360122

[pei370004-bib-0057] Hunter, D. , Borelli, T. , Beltrame, D. M. O. , Oliveira, C. N. S. , Coradin, L. , Wasike, V. W. , Wasilwa, L. , Mwai, J. , Manjella, A. , Samarasinghe, G. W. L. , Madhujith, T. , Nadeeshani, H. V. H. , Tan, A. , Ay, S. T. , Güzelsoy, N. , Lauridsen, N. , Gee, E. , & Tartanac, F. (2019). The potential of neglected and underutilized species for improving diets and nutrition. Planta, 250(3), 709–729. 10.1007/s00425-019-03169-4 31025196

[pei370004-bib-0058] Huxley, A. (1992). The new Royal Horticultural Society dictionary of gardening (Vol. 4). Macmillan Press.

[pei370004-bib-0059] Inman, E. N. , Hobbs, R. J. , & Tsvuura, Z. (2020). No safety net in the face of climate change: The case of pastoralists in Kunene Region, Namibia. PLoS ONE, 15(9), e0238982. 10.1371/journal.pone.0238982 32931518 PMC7491742

[pei370004-bib-0060] IPCC . (2019). Climate change and land: An IPCC special report on climate change, desertification, land degradation, sustainable land management, food security, and greenhouse gas fluxes in terrestrial ecosystems (P. R. Shukla, J. Skea, E. Calvo Buendia, V. Masson‐Delmotte, H.‐O. Pörtner, D. C. Roberts, P. Zhai, R. Slade, S. Connors, R. van Diemen, M. Ferrat, E. Haughey, S. Luz, S. Neogi, M. Pathak, J. Petzold, J. Portugal Pereira, P. Vyas, E. Huntley, … J. Malley, Eds.). IPCC. www.ipcc.ch

[pei370004-bib-0061] Jagermeyr, J. , Robock, A. , Elliott, J. , Muller, C. , Xia, L. , Khabarov, N. , Folberth, C. , Schmid, E. , Liu, W. , Zabel, F. , Rabin, S. S. , Puma, M. J. , Heslin, A. , Franke, J. , Foster, I. , Asseng, S. , Bardeen, C. G. , Toon, O. B. , & Rosenzweig, C. (2020). A regional nuclear conflict would compromise global food security. Proceedings of the National Academy of Sciences of the United States of America, 117(13), 7071–7081. 10.1073/pnas.1919049117 32179678 PMC7132296

[pei370004-bib-0062] Jinga, P. , Zingoni, E. , Bobo, E. D. , & Munosiyei, P. (2022). Marula (*Sclerocarya birrea* subsp. *caffra*, Anacardiaceae) thrives under climate change in sub‐Saharan Africa. African Journal of Ecology, 60(3), 736–749. 10.1111/aje.12943

[pei370004-bib-0063] Junsi, M. , & Siripongvutikorn, S. (2022). Development of herbal juice from *Centella asiatica*: Antioxidant property, nutritional value and shelf life of product. Food Science and Technology Brazil, 42, e93722. 10.1590/FST.93722

[pei370004-bib-0064] Karani, S. , Njuguna, J. , Runo, S. , Muchugi, A. , Machua, J. , & Mwaniki, P. (2022). Molecular and morphological identification of fungi causing canker and dieback diseases on *Vangueria infausta* (Burch) subsp. *rotundata* (Robyns) and *Berchemia discolor* (Klotzsch) Hemsl in lower eastern Kenya. African Journal of Biotechnology, 21(1), 6–15. 10.5897/ajb2020.17297

[pei370004-bib-0065] Keding, G. B. , Schneider, K. , & Jordan, I. (2013). Production and processing of foods as core aspects of nutrition‐sensitive agriculture and sustainable diets. Food Security, 5(6), 825–846. 10.1007/s12571-013-0312-6

[pei370004-bib-0066] Khan, M. A. , Sulemane, A. E. , Guiamba, I. R. F. , & Bechel, P. L. (2020). Processing fruits of *Vangueria infausta* (maphilwa) to obtain intermediate moisture products. Acta Horticulturae, 1292, 97–102. 10.17660/ACTAHORTIC.2020.1292.13

[pei370004-bib-0067] Khan, M. R. , & Arif, M. Z. U. (2023). Systematic review of disruptive innovation (DI) research in agriculture and future direction of research. Telematics and Informatics Reports, 11, 100079. 10.1016/j.teler.2023.100079

[pei370004-bib-0068] Khumalo, S. , Chirwa, P. W. , Moyo, B. H. , & Syampungani, S. (2012). The status of agrobiodiversity management and conservation in major agroecosystems of Southern Africa. Agriculture, Ecosystems and Environment, 157, 17–23. 10.1016/j.agee.2012.01.028

[pei370004-bib-0069] Lekoba, B. , Maluleke, M. K. , & Mphahlele, R. (2024). Nutritional composition of wild sour plum (*Ximenia caffra* subs *caffra*) fruit harvested in varying geographical regions and its potential role in human nutrition. Discover Applied Sciences, 6, 214. 10.1007/s42452-024-05874-6

[pei370004-bib-0070] Mabhaudhi, T. , Chibarabada, T. , & Modi, A. (2016). Water‐food‐nutrition‐health nexus: Linking water to improving food, nutrition and health in Sub‐Saharan Africa. International Journal of Environmental Research and Public Health, 13, 107. 10.3390/ijerph13010107 26751464 PMC4730498

[pei370004-bib-0071] Mabhaudhi, T. , Chibarabada, T. P. , Chimonyo, V. G. P. , Murugani, V. G. , Pereira, L. M. , Sobratee, N. , Govender, L. , Slotow, R. , & Modi, A. T. (2019). Mainstreaming underutilized indigenous and traditional crops into food systems: A South African perspective. Sustainability, 11, 172. 10.3390/su11010172 PMC7615043 37681213

[pei370004-bib-0072] Mabhaudhi, T. , Chimonyo, V. G. P. , Chibarabada, T. P. , & Modi, A. T. (2017). Developing a roadmap for improving neglected and underutilized crops: A case study of South Africa. Frontiers in Plant Science, 8, 2143. 10.3389/fpls.2017.02143 29312397 PMC5735103

[pei370004-bib-0073] Mabhaudhi, T. , Chimonyo, V. G. P. , & Modi, A. T. (2017). Status of underutilised crops in South Africa: Opportunities for developing research capacity. Sustainability, 9, 1569. 10.3390/su9091569

[pei370004-bib-0074] Mabhaudhi, T. , O'Reilly, P. , Walker, S. , & Mwale, S. (2016). Opportunities for underutilised crops in Southern Africa's post‐2015 development agenda. Sustainability, 8, 302. 10.3390/su8040302

[pei370004-bib-0075] Magaia, T. , Uamusse, A. , Sjöholm, I. , & Skog, K. (2013). Proximate analysis of five wild fruits of Mozambique. The Scientific World Journal, 2013, 601435. 10.1155/2013/601435 23983641 PMC3745983

[pei370004-bib-0076] Maina, S. , Ryu, D. H. , Bakari, G. , Misinzo, G. , Nho, C. W. , & Kim, H. Y. (2021). Variation in phenolic compounds and antioxidant activity of various organs of African cabbage (*Cleome gynandra* L.) accessions at different growth stages. Antioxidants, 10(12), 1952. 10.3390/antiox10121952 34943055 PMC8750509

[pei370004-bib-0077] Makaza, W. , Gasura, E. , Nyakurwa, C. S. , & Masekesa, R. T. (2022). Prospects in cultivation and utilization of spiderplant (*Cleome gynandra* L.) in sub‐Saharan Africa: A review. African Journal of Food, Agriculture, Nutrition and Development, 22(1), 19370–19385. 10.18697/ajfand.106.20040

[pei370004-bib-0078] Malebana, I. M. M. , Nkosi, B. D. , Erlwanger, K. H. , & Chivandi, E. (2018). A comparison of the proximate, fibre, mineral content, amino acid and the fatty acid profile of Marula (*Sclerocarya birrea caffra*) and soyabean (*Glycine max*) meals. Journal of the Science of Food and Agriculture, 98(4), 1381–1387. 10.1002/jsfa.8604 28758208

[pei370004-bib-0079] Marshall, E. , Newton, A. C. , & Schreckenberg, K. (2003). Commercialisation of non‐timber forest products: First steps in analysing the factors influencing success. International Forestry Review, 5(2), 128–137.

[pei370004-bib-0080] Mashau, M. E. , Kgatla, T. E. , Makhado, M. V. , Mikasi, M. S. , & Ramashia, S. E. (2022). Nutritional composition, polyphenolic compounds and biological activities of marula fruit (*Sclerocarya birrea*) with its potential food applications: A review. International Journal of Food Properties, 25(1), 1549–1575. 10.1080/10942912.2022.2064491

[pei370004-bib-0081] Mashilo, J. , Odindo, A. O. , Shimelis, H. A. , Musenge, P. , Tesfay, S. Z. , & Magwaza, L. S. (2017). Drought tolerance of selected bottle gourd [*Lagenaria siceraria* (Molina) Standl.] landraces assessed by leaf gas exchange and photosynthetic efficiency. Plant Physiology and Biochemistry, 120, 75–87. 10.1016/j.plaphy.2017.09.022 28988036

[pei370004-bib-1003] Maxted, N. , & Vincent, H. (2021). Review of congruence between global crop wild relative hotspots and centres of crop origin/diversity. In Genetic resources and crop evolution (Vol. 68, Issue 4, pp. 1283–1297). Springer Science and Business Media B.V. 10.1007/s10722-021-01114-7

[pei370004-bib-0082] Mbhele, Z. , Zharare, G. E. , Zimudzi, C. , Mchunu, C. N. , & Ntuli, N. R. (2024). Variation in nutritional composition of *Strychnos spinosa* Lam. morphotypes in KwaZulu‐Natal, South Africa. Genetic Resources and Crop Evolution. 10.1007/s10722-024-01982-9

[pei370004-bib-0083] Mbhele, Z. , Zharare, G. E. , Zimudzi, C. , & Ntuli, N. R. (2023). Assessing genetic variation among *Strychnos spinosa* Lam. morphotypes using simple sequence repeat markers. Plants, 12, 2810. 10.3390/plants12152810 37570964 PMC10421500

[pei370004-bib-0084] Meda, R. S. , Jain, S. , Singh, S. , Verma, C. , Nandi, U. , & Maji, P. K. (2022). Novel *Lagenaria siceraria* peel waste based cellulose nanocrystals: Isolation and rationalizing H‐bonding interactions. Industrial Crops and Products, 186, 115197. 10.1016/j.indcrop.2022.115197

[pei370004-bib-0085] Mertz, C. , Ranovona, Z. , Dhuique‐Mayer, C. , Servent, A. , Dornier, M. , Danthu, P. , & Ralison, C. (2019). The nutrient content of two folia morphotypes of *Centella asiatica* (L) grown in Madagascar. African Journal of Food Agriculture Nutrition and Development, 19(3), 14654–14673. 10.18697/ajfand.86.17750

[pei370004-bib-0086] Mondal, M. , Biswas, P. P. , & De, S. (2016). Clarification and storage study of bottle gourd (*Lagenaria siceraria*) juice by hollow fiber ultrafiltration. Food and Bioproducts Processing, 100, 1–15. 10.1016/j.fbp.2016.06.010

[pei370004-bib-0087] Moyo, M. , & Aremu, A. O. (2022). Nutritional, phytochemical and diverse health‐promoting qualities of *Cleome gynandra* . Critical Reviews in Food Science and Nutrition, 62(13), 3535–3552. 10.1080/10408398.2020.1867055 33397131

[pei370004-bib-0088] Muetzel, S. , Hoffmann, E. M. , & Becker, K. (2003). Supplementation of barley straw with *Sesbania pachycarpa* leaves in vitro: Effects on fermentation variables and rumen microbial population structure quantified by ribosomal RNA‐targeted probes. British Journal of Nutrition, 89(4), 445–453. 10.1079/bjn2002813 12654162

[pei370004-bib-0089] Mukwada, G. , Mazibuko, S. M. , Moeletsi, M. , & Robinson, G. M. (2021). Can famine be averted? A spatiotemporal assessment of the impact of climate change on food security in the Luvuvhu River catchment of South Africa. Landscape, 10, 527. 10.3390/land10050527

[pei370004-bib-0090] Muok, B. O. , & Ishii, T. (2006). Effect of Arbuscular Mycorrhizal fungi on tree growth and nutrient uptake of *Sclerocarya birrea* under water stress, salt stress and flooding. Society for Horticultural Science, 75(1), 26–31.

[pei370004-bib-0091] Narendhirakannan, R. T. , Subramanian, S. , & Kandaswamy, M. (2007). Anti‐inflammatory and lysosomal stability actions of *Cleome gynandra* L. studied in adjuvant induced arthritic rats. Food and Chemical Toxicology, 45(6), 1001–1012. 10.1016/j.fct.2006.12.009 17276570

[pei370004-bib-0092] Ndhlala, A. R. , Chitindingu, K. , Mupure, C. , Murenje, T. , Ndhlala, F. , Benhura, M. A. , & Muchuweti, M. (2008). Antioxidant properties of methanolic extracts from *Diospyros mespiliformis* (jackal berry), *Flacourtia indica* (Batoka plum), *Uapaca kirkiana* (wild loquat) and *Ziziphus mauritiana* (yellow berry) fruits. International Journal of Food Science and Technology, 43(2), 284–288. 10.1111/j.1365-2621.2006.01431.x

[pei370004-bib-0093] Ndhlala, A. R. , Muchuweti, M. , Mupure, C. , Chitindingu, K. , Murenje, T. , Kasiyamhuru, A. , & Benhura, M. A. (2008). Phenolic content and profiles of selected wild fruits of Zimbabwe: *Ximenia caffra*, *Artobotrys brachypetalus* and *Syzygium cordatum* . International Journal of Food Science and Technology, 43(8), 1333–1337. 10.1111/j.1365-2621.2007.01611.x

[pei370004-bib-0094] Neumann, R. P. , & Hirsch, E. (2000). Commercialisation of non‐timber forest products: Review and analysis of research. Center for International Forestry Research (CIFOR). 10.17528/cifor/000723

[pei370004-bib-0095] Neves, M. , Antunes, M. , Fernandes, W. , Campos, M. J. , Azevedo, Z. M. , Freitas, V. , Rocha, J. M. , & Tecelão, C. (2021). Physicochemical and nutritional profile of leaves, flowers, and fruits of the edible halophyte chorão‐da‐praia (*Carpobrotus edulis*) on Portuguese west shores. Food Bioscience, 43, 101288. 10.1016/j.fbio.2021.101288

[pei370004-bib-0096] Nhamo, L. , Mabhaudhi, T. , & Modi, A. T. (2019). Preparedness or repeated short‐term relief aid? Building drought resilience through early warning in southern Africa. Water SA, 45(1), 75–85. 10.4314/wsa.v45i1.09

[pei370004-bib-0097] Nhukarume, L. , Chikwambi, Z. , Muchuweti, M. , & Chipurura, B. (2010). Phenolic content and antioxidant capacities of *Parinari curatelifolia*, *Strychnos spinosa* and *Adansonia digitata* . Journal of Food Biochemistry, 34(SUPPL. 1), 207–221. 10.1111/j.1745-4514.2009.00325.x

[pei370004-bib-0098] Nkosi, N. N. , Mostert, T. H. C. , Dzikiti, S. , & Ntuli, N. R. (2020). Prioritization of indigenous fruit tree species with domestication and commercialization potential in KwaZulu‐Natal, South Africa. Genetic Resources and Crop Evolution, 67(6), 1567–1575. 10.1007/s10722-020-00932-5

[pei370004-bib-0099] Nyoka, B. I. , Chanyenga, T. , Mng'omba, S. A. , Akinnifesi, F. K. , & Sagona, W. (2015). Variation in growth and fruit yield of populations of *Sclerocarya birrea* (A. Rich.) Hochst. Agroforestry Systems, 89, 397–407. 10.1007/s10457-014-9774-6

[pei370004-bib-0100] Odhiambo, F. , Id, O. , Boedecker, J. , & Kennedy, G. (2019). Exploring agrobiodiversity for nutrition: Household on‐farm agrobiodiversity is associated with improved quality of diet of young children in Vihiga, Kenya. PLoS One, 14(8), e0219680. 10.1371/journal.pone.0219680 31374090 PMC6677318

[pei370004-bib-0101] Odhiambo, O. V. (2020). *Conditions for optimum germination of sprawling Bauhinia seed* (*Tylosema fassoglense*) *(Kotschy ex Schweinf.) Torre & Hillc* [Master of Science]. University of Nairobi.

[pei370004-bib-0102] Ogunbusola, E. M. (2018). Nutritional and antinutritional composition of calabash and bottle gourd seed flours (var *Lagenaria siceraria*). Journal of Culinary Science & Technology, 16(4), 326–335. 10.1080/15428052.2017.1390518

[pei370004-bib-0103] Olajuyigbe, S. O. , Jimoh, S. O. , Adegeye, A. O. , & Mukhtar, R. B. (2012). Drought stress on early growth of *Diospyros mespiliformis* Hochst ex A. Rich in Jega, Northern Nigeria. Nigerian Journal of Ecology, 12, 71–76.

[pei370004-bib-0104] Omondi, E. O. , Engels, C. , Nambafu, G. , Schreiner, M. , Neugart, S. , Abukutsa‐Onyango, M. , & Winkelmann, T. (2017). Nutritional compound analysis and morphological characterization of spider plant (*Cleome gynandra*)—An African indigenous leafy vegetable. Food Research International, 100, 284–295. 10.1016/j.foodres.2017.06.050 28873690

[pei370004-bib-0105] Omotayo, A. O. , & Aremu, A. O. (2021). Undervalued spiny monkey orange (*Strychnos spinosa* lam.): An indigenous fruit for sustainable food‐nutrition and economic prosperity. Plants, 10(12), 2785. 10.3390/plants10122785 34961256 PMC8703348

[pei370004-bib-0106] Oosthuizen, D. , Goosen, N. J. , Stander, M. A. , Ibrahim, A. D. , Pedavoah, M. M. , Usman, G. O. , & Aderinola, T. (2018). Solvent extraction of polyphenolics from the indigenous African fruit *Ximenia caffra* and characterization by LC‐HRMS. Antioxidants, 7(8), 103. 10.3390/antiox7080103 30071585 PMC6116166

[pei370004-bib-0107] Ord, T. (2020). The recipice: Existential risk and the future of humanity. Hachette Books.

[pei370004-bib-0108] Orimoloye, I. R. , Zhou, L. , & Kalumba, A. M. (2021). Drought disaster risk adaptation through ecosystem services‐based solutions: Way forward for South Africa. Sustainability, 13, 4132. 10.3390/su13084132

[pei370004-bib-0109] Ortiz, R. (2011). Agrobiodiversity management for climate change. In J. M. Lenn'e & D. Wood (Eds.), Agrobiodiversity management for food security: A critical review (pp. 189–211). CABI.

[pei370004-bib-0110] Palmer, E. , & Pitman, N. (1972). Trees of Southern Africa. Balkema.

[pei370004-bib-0111] Paumgarten, F. , Locatelli, B. , & Witkowski, E. T. F. (2018). Wild foods: Safety net or poverty trap? A South African case study. Human Ecology, 46(2), 183–195. 10.1007/s10745-018-9984-z

[pei370004-bib-0112] Pereira, C. G. , Neng, N. R. , & Custódio, L. (2023). From threat to opportunity: Harnessing the invasive *Carpobrotus edulis* (L.) N.E.Br for nutritional and phytotherapeutic valorization amid seasonal and spatial variability. Marine Drugs, 21, 436. 10.3390/md21080436 37623717 PMC10456270

[pei370004-bib-0113] Piñeiro, V. , Arias, J. , Dürr, J. , Elverdin, P. , Ibáñez, A. M. , Kinengyere, A. , Opazo, C. M. , Owoo, N. , Page, J. R. , Prager, S. D. , & Torero, M. (2020). A scoping review on incentives for adoption of sustainable agricultural practices and their outcomes. Nature Sustainability, 3(10), 809–820. 10.1038/s41893-020-00617-y

[pei370004-bib-0114] Poochi, S. P. , Easwaran, M. , Balasubramanian, B. , Anbuselvam, M. , Meyyazhagan, A. , Park, S. , Bhotla, H. K. , Anbuselvam, J. , Arumugam, V. A. , Keshavarao, S. , Kanniyappan, G. V. , Pappusamy, M. , & Kaul, T. (2020). Employing bioactive compounds derived from *Ipomoea obscura* (L.) to evaluate potential inhibitor for SARS‐CoV‐2 main protease and ACE2 protein. Food Frontiers, 1(2), 168–179. 10.1002/fft2.29 32838301 PMC7361879

[pei370004-bib-0115] Priyanka, S. P. , Sujatha, S. , Smitha, G. R. , Suryanarayana, M. A. , & Kalaivana, D. (2022). Biomass accumulation, bioactive compounds and nutrient uptake in *Centella asiatica* (L.) in relation to organic nutrition in open‐field and shade. Industrial Crops and Products, 176, 114352. 10.1016/j.indcrop.2021.114352

[pei370004-bib-0116] Ramadwa, T. E. , & Meddows‐Taylor, S. (2023). Traditional uses, pharmacological activities, and phytochemical analysis of *Diospyros mespiliformis* Hochst. ex. A. DC (Ebenaceae): A review. Molecules, 28(23), 7759. 10.3390/molecules28237759 38067488 PMC10708241

[pei370004-bib-0117] Rosero, A. , Granda, L. , Berdugo‐Cely, J. A. , Šamajová, O. , Šamaj, J. , & Cerkal, R. (2020). A dual strategy of breeding for drought tolerance and introducing drought‐tolerant, underutilized crops into production systems to enhance their resilience to water deficiency. Plants, 9, 1263. 10.3390/plants9101263 32987964 PMC7600178

[pei370004-bib-0118] Saka, J. K. K. , & Msonthi, J. D. (1994). Nutritional value of edible fruits of indigenous wild trees in Malawi. Forest Ecology and Management, 64, 245–248.

[pei370004-bib-0119] Salami, S. O. , Adegbaju, O. D. , Idris, O. A. , Jimoh, M. O. , Olatunji, T. L. , Omonona, S. , Orimoloye, I. R. , Adetunji, A. E. , Olusola, A. , Maboeta, M. S. , & Laubscher, C. P. (2022). South African wild fruits and vegetables under a changing climate: The implications on health and economy. South African Journal of Botany, 145, 13–27. 10.1016/j.sajb.2021.08.038

[pei370004-bib-0120] Sartas, M. , Schut, M. , Proietti, C. , Thiele, G. , & Leeuwis, C. (2020). Scaling readiness: Science and practice of an approach to enhance impact of research for development. Agricultural Systems, 183, 102874. 10.1016/j.agsy.2020.102874

[pei370004-bib-0121] Sibiya, N. P. , Kayitesi, E. , & Moteetee, A. N. (2021). Proximate analyses and amino acid composition of selected wild indigenous fruits of Southern Africa. Plants, 10(4), 721. 10.3390/plants10040721 33917651 PMC8068051

[pei370004-bib-0122] Sikora, R. A. , Terry, E. R. , Vlek, P. L. , & Chitja, J. (2020). Transforming agriculture in Southern Africa; Constraints, technologies, policies and processes. Taylor & Francis.

[pei370004-bib-0123] Sitrit, Y. , Loison, S. , Ninio, R. , Dishon, E. , Bar, E. , Lewinsohn, E. , & Mizrahi, Y. (2003). Characterization of monkey orange (*Strychnos spinosa* Lam.), a potential new crop for arid regions. Journal of Agricultural and Food Chemistry, 51(21), 6256–6260. 10.1021/jf030289e 14518952

[pei370004-bib-0124] Smith, G. S. , Anjum, E. , Francis, C. , Deanes, L. , & Acey, C. (2022). Climate change, environmental disasters, and health inequities: The underlying role of structural inequalities. Current Environmental Health Reports, 9, 80–89. 10.1007/s40572-022-00336-w 35338470

[pei370004-bib-0125] Tamariz, G. (2022). Geoforum agrobiodiversity conservation with illegal‐drug crops: An approach from the prisons in Oaxaca, Mexico. Geoforum, 128, 300–311. 10.1016/j.geoforum.2020.10.012

[pei370004-bib-0126] Temoso, O. , Hadley, D. , & Villano, R. (2018). Sources of efficiency, productivity and output growth in Botswana agriculture. Review of Development Economics, 22(3), 1105–1124. 10.1111/rode.12376

[pei370004-bib-0127] Temple, V. J. , Onobun, C. E. , & Ojobe, T. O. (1991). Chemical composition of livingstone potato tubers (*Plectranthus esculentus*). Journal of the Science of Food and Agriculture, 56(2), 215–217. 10.1002/jsfa.2740560210

[pei370004-bib-0128] The World Bank . (2023). World development indicators. The World Bank.

[pei370004-bib-0129] Thornton, P. , Mason D'Croz, D. , Kugler, C. , Remans, R. , Zornetzer, H. , & Herrero, M. (2024). Enabling food system innovation: Accelerators for change. Global Food Security, 40, 100738. 10.1016/j.gfs.2023.100738 38567265 PMC10983825

[pei370004-bib-0130] Toon, O. B. , Bardeen, C. G. , Robock, A. , Xia, L. , Kristensen, H. , McKinzie, M. , Peterson, R. J. , Harrison, C. S. , Lovenduski, N. S. , & Turco, R. P. (2019). Rapidly expanding nuclear arsenals in Pakistan and India portend regional and global catastrophe. Science Advances, 5(10), 1–14. 10.1126/sciadv.aay5478 PMC6774726 31616796

[pei370004-bib-0131] Toon, O. B. , Robock, A. , Turco, R. P. , Bardeen, C. , Oman, L. , & Stenchikov, G. L. (2007). Consequences of regional‐scale nuclear conflicts. Science, 315(5816), 1224–1225. 17332396 10.1126/science.1137747

[pei370004-bib-0132] van den Heever, E. , & Venter, S. L. (2007). Nutritional and medicinal properties of *Cleome gynandra* . Acta Horticulturae, 752, 127–130.

[pei370004-bib-0133] van Rayne, K. K. , Adebo, O. A. , Wokadala, O. C. , & Ngobese, N. Z. (2023). The potential of *Strychnos* spp L. Utilization in food insecurity alleviation: A review. Food Reviews International, 39(6), 3400–3414. 10.1080/87559129.2021.2012791

[pei370004-bib-0134] van Zonneveld, M. , Kindt, R. , McMullin, S. , Achigan‐Dako, E. G. , N'Danikou, S. , Hsieh, W. H. , Lin, Y. R. , & Dawson, I. K. (2023). Forgotten food crops in sub‐Saharan Africa for healthy diets in a changing climate. Proceedings of the National Academy of Sciences of the United States of America, 120(14), e2205794120. 10.1073/pnas.2205794120 36972432 PMC10083591

[pei370004-bib-0135] Verma, A. K. , Sharma, B. D. , & Banerjee, R. (2012). Quality characteristics of low‐fat chicken nuggets: Effect of common salt replacement and added bottle gourd (*Lagenaria siceraria* L.). Journal of the Science of Food and Agriculture, 92(9), 1848–1854. 10.1002/jsfa.5691 22511275

[pei370004-bib-0136] Waldman, K. B. , Giroux, S. , Blekking, J. P. , Baylis, K. , & Evans, T. P. (2020). Smallholder food storage dynamics and resilience. Food Security, 12(1), 7–20. 10.1007/s12571-019-00983-2

[pei370004-bib-0137] Welcome, A. K. , & van Wyk, B. E. (2019). An inventory and analysis of the food plants of southern Africa. South African Journal of Botany, 122, 136–179. 10.1016/j.sajb.2018.11.003

[pei370004-bib-0138] Williams, J. T. , & Haq, N. (2000). Global research on underutilised crops an assessment of current activities and proposals for enhanced cooperation. International Centre for Underutilized Crops.

[pei370004-bib-0139] Winstead, D. J. , & Jacobson, M. G. (2022a). Food resilience in a dark catastrophe: A new way of looking at tropical wild edible plants. Ambio, 51, 1949–1962. 10.1007/s13280-022-01715-1 35290618 PMC9287517

[pei370004-bib-0140] Winstead, D. J. , & Jacobson, M. G. (2022b). Forest resource availability after nuclear war or other sun‐blocking catastrophes. Earth's Future, 10(7), e2021EF002509. 10.1029/2021EF002509

[pei370004-bib-0141] Winstead, D. J. , Jacobson, M. G. , & Di Gioia, F. (2023). Valorizing staple Native American food plants as a food resilience resource. Frontiers in Sustainable Food Systems, 7, 1117805. 10.3389/fsufs.2023.1117805

[pei370004-bib-0142] Wongfhun, P. , Gordon, M. H. , & Apichartsrangkoon, A. (2010). Flavour characterisation of fresh and processed pennywort (*Centella asiatica* L.) juices. Food Chemistry, 119, 69–74. 10.1016/j.foodchem.2009.05.072 20492095

[pei370004-bib-0143] Xia, L. , Robock, A. , Mills, M. , Stenke, A. , & Helfand, I. (2015). Decadal reduction of Chinese agriculture after a regional nuclear war. Earth's Future, 3(2), 37–48. 10.1002/2014EF000283

